# Real world testing and cost‐effectiveness analysis of subcutaneous EEG (REAL‐ASE): Protocol for a prospective multicentre interventional trial

**DOI:** 10.1002/epi4.70064

**Published:** 2025-06-10

**Authors:** Matthew McWilliam, Pedro F. Viana, Eren Dursun, Joe Davies, Joel S. Winston, Jessica Agren, Harutomo Hasegawa, Jonas Duun‐Henriksen, Mark P. Richardson

**Affiliations:** ^1^ Institute of Psychiatry, Psychology and Neuroscience King's College London London UK; ^2^ Epilepsy Centre, King's College Hospital NHS Foundation Trust London UK; ^3^ UNEEG Medical A/S Lillerød Denmark

**Keywords:** EEG, electroencephalography, epilepsy, mobile technology, seizures, wearables

## Abstract

**Objective:**

Epilepsy is a common condition associated with significant morbidity, mortality, and costs. Poor documentation of seizures is a major challenge in epilepsy care. Objective seizure counting with mobile devices may mitigate this challenge and improve patient management. The aim of this study is to investigate whether ultra long‐term subcutaneous EEG improves seizure documentation and disease monitoring in people with drug‐resistant epilepsy.

**Methods:**

Real World Testing and Cost‐effectiveness Analysis of Subcutaneous EEG (REAL‐ASE) is a UK‐based multi‐centre prospective interventional study with an expected duration of 7 months. Thirty‐three adult participants will be implanted with 24/7 EEG SubQ and collect 2‐channel EEG data for 6 months. Data will be reviewed and annotated by experts, and a summary sent to the treating clinician weekly. The treating clinician will communicate information from this annotation report to participants monthly. Changes in management can be made at the treating clinician's discretion. REAL‐ASE was approved by the Bromley Research Ethics Committee in July 2023 (reference number 23/LO/0419).

**Results:**

We anticipate that subcutaneous EEG may improve seizure documentation and be a well‐accepted addition to epilepsy care by clinicians and patients. We believe it will be associated with better quality of life and will be a cost‐effective solution.

**Significance:**

If our study demonstrates improved seizure documentation with subcutaneous EEG, this may contribute to patient safety, improved quality of life, and a reduction in healthcare costs.

**Plain Language Summary:**

We present a clinical trial protocol for a prospective cohort study of 33 people with epilepsy across the UK. The study aims to assess whether an EEG implant placed under the skin: (1) is more accurate than patient‐reported seizure diary, (2) is feasible and acceptable to patients and clinicians, (3) reduces the impact of epilepsy, and (4) is beneficial to the National Health Service (NHS).

**Key points**

- Current guidelines emphasize regular seizure frequency assessments. Patient diaries are ubiquitous, but evidence shows they are unreliable.
- There have been no previous clinical trials to evaluate the role of a CE‐marked subcutaneous EEG device within the NHS.
- We describe a clinical trial protocol assessing the utility of two‐channel subcutaneous EEG compared to patient‐reported seizure diaries.
- This trial will evaluate whether the use of subcutaneous EEG can improve seizure frequency documentation in the outpatient setting.

## 1 INTRODUCTION

### 1.1 Background and rationale

Epilepsy is a common disorder. In the UK, approximately 550 000–800 000 people are currently being treated for epilepsy in the National Health Service (NHS).^1^, ^2^ Epilepsy is also costly, with the total direct cost in the European Union estimated at €15.5billion,^3^ particularly related to the costs of anti‐seizure medications and the costs of emergency admissions.^4^ Epileptic seizures are one of the commonest reasons for repeated emergency admissions in the NHS.^5^ Seizures are the key symptom of epilepsy and the most important predictor of quality of life.^6^ Approximately 30% of people with epilepsy have seizures despite being on apparently optimal treatment.^7^ Tragically, epilepsy is the 5th leading cause of avoidable years of life lost in males, and the 8th leading cause in females,^8^ with most of these deaths directly attributable to the consequences of seizures.

The majority (60%–68%) of people with epilepsy experience focal seizures.^9^, ^10^, ^11^, ^12^ A recent meta‐analysis revealed focal‐onset seizures conferred a relative risk of 3.36 for treatment resistance.^13^

In clinical practice, a participant‐reported seizure diary is the ubiquitous outcome measure to evaluate the effectiveness of anti‐seizure medications.^14^ There is strong evidence that participant‐reported seizure diaries are unreliable. For example, using in‐hospital video‐EEG as the ground truth for seizure documentation, studies found 44%–56% of seizures were not reported.^15^, ^16^, ^17^ Reasons for under‐reporting seizures might include unawareness that a seizure has occurred, post‐ictal amnesia, seizures occurring during sleep, uncertainty about the number of seizures occurring during a cluster, and incomplete adherence to keeping a diary. In a study using a device recording intracranial EEG in 11 participants for several months, the average correlation between monthly diary seizure counts and monthly ground truth intracranial EEG‐detected seizure counts was weak, only 0.30.^18^ Poor seizure documentation may lead to suboptimal management. Patients who fail to report seizures are at increased risk of seizure‐related harm, and patients who over‐report certain symptoms as seizures may be at risk of excessive treatment side effects.

The need for more objective seizure monitoring has led to the development of several mobile health technologies.^19^, ^20^ To date, most are approved for the detection of generalized or focal‐to‐bilateral tonic–clonic seizures. Furthermore, there is very limited real‐world evidence to support the reliability of most devices, as most evidence is generated from recordings in the hospital environment or in supervised care settings. Hence, there is a critical gap for a portable, discreet device able to detect a wider range of seizure types, particularly focal seizures.

**FIGURE 1:**
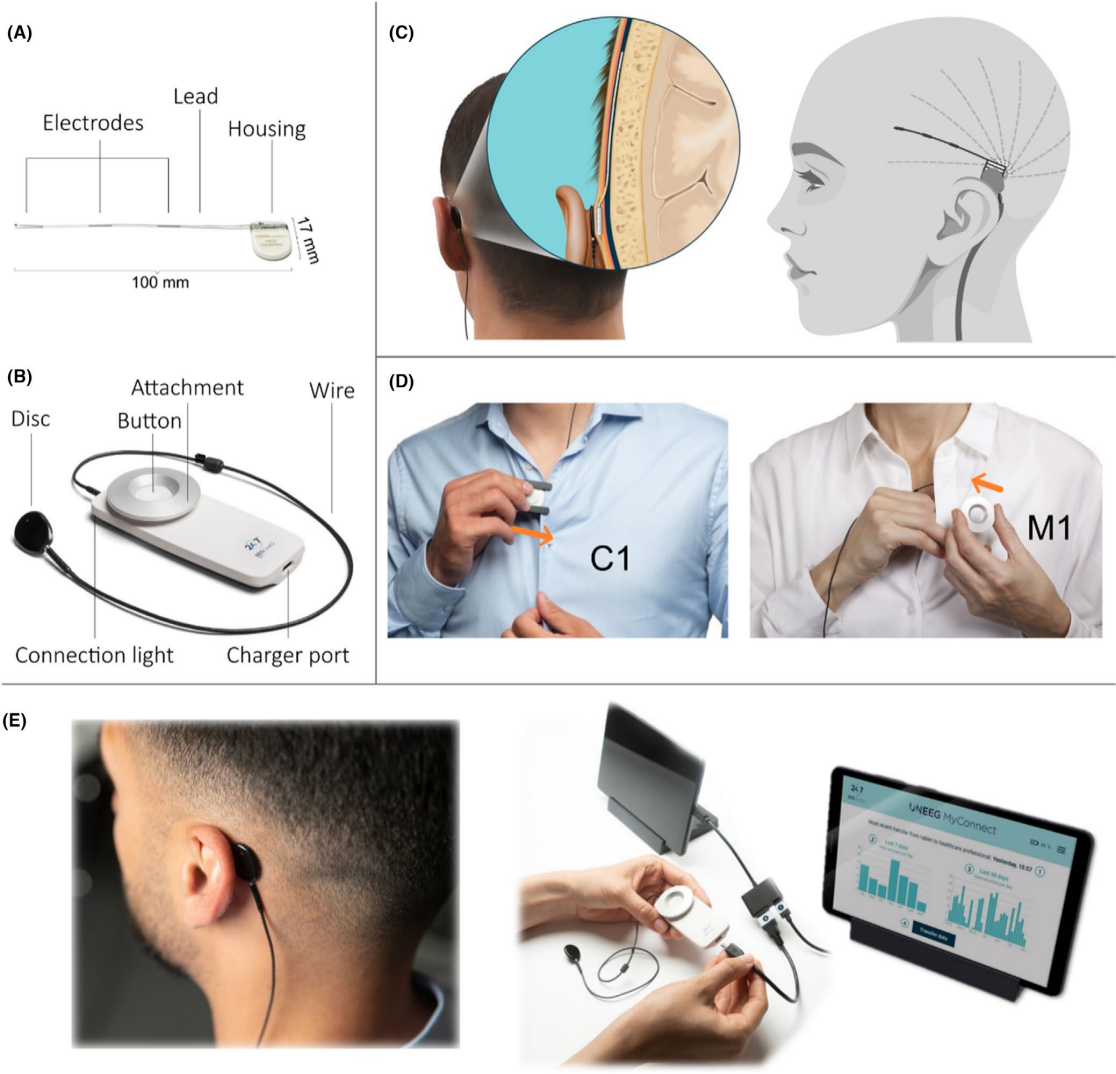
Main components of the 24/7 EEG SubQ solution (UNEEG medical). (A) The implant consists of a lead with three electrodes and a ceramic housing with the electronics. (B) The external device powers the implant and enables recording of the data. (C) The implant is implanted entirely subcutaneously. (D) The device attaches to the clothes via a plastic clip (C1) or a disc‐shaped magnet. (E) For recording, the wire and disc of the external device attach to the skin overlying the implant housing with a small adhesive patch. Once a day, the external device is attached to a tablet for charging and transferring the data via a cloud‐based solution to the healthcare professional (HCP).

There has been rapid development of EEG‐based mobile technologies, such as subcutaneous EEG. To date, one device has received CE mark for ultra long‐term ambulatory EEG recording—the 24/7 EEG SubQ system (UNEEG Medical; Allerød, Denmark) illustrated in Figure 1.^21^

Using the 24/7 EEG SubQ system, it is feasible to record EEG data reliably from home for up to 15 months.^22^, ^23^ Preliminary data suggest the device has recording characteristics similar to scalp EEG (for 2 bipolar channels),^24^ with signal quality showing high long‐term stability.^22^ Observational studies have suggested its utility for objective seizure counting, including the detection of temporal periodicities of seizure occurrence.^25^, ^26^ However, it remains uncertain whether this tool is of demonstrated added value when used prospectively, in the real world.

### 1.2 Objectives

The primary objectives for this study are to investigate whether 24/7 EEG SubQ, (1) is more informative than a participant‐reported seizure diary, (2) is feasible and acceptable to participants and clinicians, (3) reduces the impacts of epilepsy and improves quality of life, and (4) provides gains to the healthcare system when rolled out into the NHS.

## 2 METHODS

### 2.1 Trial design

REAL‐ASE (Clinical Trials NCT06144047) is a prospective, non‐randomized, multicentre, interventional study that will be performed across UK neurology centres. A total of 33 adults with epilepsy, treated as part of their routine clinical care across the recruitment sites, will be enrolled.

### 2.2 Study setting

The study will take place in UK hospitals with integrated neurology centres, recruiting participants as outpatients.

### 2.3 Eligibility criteria

Inclusion criteria are: (1) given written informed consent; (2) adults (≥18 years of age); (3) routinely keep a seizure diary, have access to a smartphone, and are willing to use the electronic diary for the study; (4) experiencing 10 or more seizures per year according to the seizure diary; (5) have had an epilepsy diagnosis for at least 2 years; and (6) willing to comply with study procedures.

Exclusion criteria are: (1) cochlear implant(s); (2) established current diagnosis of psychogenic non‐epileptic attacks (dissociative seizures); (3) frequent vigorous involuntary movements (e.g. chorea, athetosis) or frequent parasomnias with major motor components (e.g. sleep walking, night terrors); (4) participants involved in therapies with medical devices that deliver electrical/radiofrequency energy into the area around the implant; (5) participants at high risk of surgical complications, such as active systemic infection and haemorrhagic disease; (6) allergy to the local anesthetics used during implantation; (7) infection at the site of device implantation; (8) participants who operate MRI scanners or are planned to have an MRI scan during the study period; (9) participants with profession/hobby that includes activity imposing extreme pressure variations (e.g. diving or parachute jumping). NB: diving/snorkeling is allowed to 5 meters of depth; (10) participants with profession/hobby that includes activity imposing an unacceptable risk for trauma against the device or the site of implantation (e.g. martial art or boxing); and (11) Any other serious medical condition that in the opinion of the Chief Investigator would be incompatible with participation in the study.

### 2.4 Sample size

We powered our study based on the primary outcome measure for diagnostic accuracy. We used data from an intracranial EEG study,^18^ which shows the correlation between the monthly number of seizure occurrences documented using participant self‐reported diary and the ground truth seizure occurrences per month, documented with the intracranial Neurovista device. The mean correlation across the group is 0.30 (SD 0.41). We seek to demonstrate a higher correlation between 24/7 EEG SubQ and the ground truth seizure occurrences. We chose a modestly higher correlation of 0.5 to power this study. At alpha 0.05, 33 participants would give 85% power to demonstrate an improvement in within‐participants correlation from 0.3 to 0.5 (calculated using G*Power).

### 2.5 Recruitment

People with epilepsy (PWE) will be identified by investigators through epilepsy clinic attendance lists, electronic patient records, and hospital Epilepsy Monitoring Unit admission lists at the participating sites. Potential participants will be approached by telephone, email, letter or in clinic by an investigator. This contact will be designated the ‘first approach’ and will involve providing the participant information leaflet. Subsequently, after at least 24 hours, potential participants will be invited to a face‐to‐face meeting (Visit 1) during which they will be given the opportunity to discuss the study. If they opt to enroll in the study, they will provide informed consent to participate.

## 3 STUDY PROCEDURES

During Visit 1, participants will complete baseline patient reported outcome measures (PROMs) and subsequently will undergo an online qualitative interview at baseline. Following this, they will be implanted with the 24/7 EEG SubQ by the local surgical team (Visit 2). The implant is inserted under local anesthesia in the pre‐auricular region in the subgaleal space on a predetermined side (i.e. right or left) and orientation based on the treating clinician's instruction. The electrodes can be positioned anywhere in the arc illustrated on the right of Figure 1 panel C, which allows a wide range of focal epilepsies to be included. We aim to position the electrodes where ictal EEG changes are likely to be detected based on previous electroclinical data. Any surgical specialty with expertise operating within the head and neck region is eligible to implant the 24/7 EEG SubQ. The participant is discharged after a two‐hour observation period with an implant ID card. Visit 3 occurs 10–14 days after implantation, when procedures for data collection will again be explained, and the participant will commence EEG data collection and begin keeping an electronic seizure diary (Helpilepsy; Neuroventis, Overijse, Belgium).^27^ During 6 months of data collection, participants will receive a minimum of monthly feedback regarding recorded EEG data from their treating clinician. They will repeat PROMs at 2 months (Visit 4) and 6 months (Visit 5) and an online qualitative interview prior to explantation after data collection is complete. The participant will then exit the study. Treating clinicians will complete standardized questionnaires assessing their experience using the 24/7 EEG SubQ during the study and after the participant's last visit. A treating clinician at each site will participate in a qualitative interview after the last participant at that site has had his/her final visit.

**FIGURE 2:**
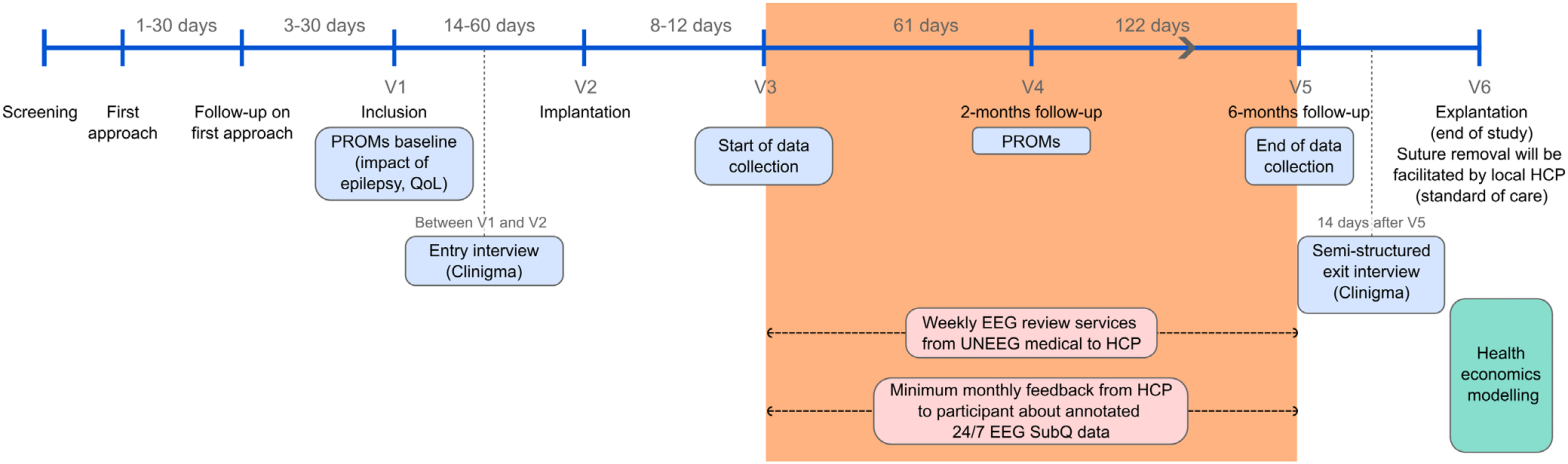
Study schedule.

The study investigators will provide careful and detailed explanation about study procedures (including follow up plans) during the first approach, follow up approach, and Visit 1. Participants will be reminded of study visits prior to their occurrence. Participants will be provided with study point of contact details (Study Co‐ordinator and Clinical Research Fellow) and will be given ample notice for visit dates and times. There will be an option to complete Visits 4 and 5 remotely by phone if this is more convenient. Clinician monthly feedback to participants can be given through any preferred medium, as agreed between participant and clinician. See Figure 2 for complete study schedule.

### 3.1 Data collection

PROMs will be collected at Visits 1 (baseline), 4 (2 months), and 5 (6 months) via the electronic data capture system REDCap (see Table 1 for details).^28^ Use of REDCap forced responses to progress surveys and email prompts to review data will be utilized to ensure data completeness with both participants and investigators.

**TABLE 1:** Primary and secondary objectives with corresponding outcome measures.

Primary objective	Corresponding endpoint(s)/outcome variable(s)
To investigate the diagnostic accuracy of 24/7 EEG™ SubQ Solution	Within‐participant correlation between seizure counts per month estimated from rapid clinician review of annotated 24/7 EEG™ SubQ data and ‘ground truth’ EEG seizure occurrences per month (derived from expert research team review of 24/7 EEG™ SubQ EEG data), across the 6 months of the study (‘correlation A’). Within‐participant correlation between seizure counts per month from participant‐reported seizure diary and ‘ground truth’ EEG seizure occurrences per month (derived from expert research team review of 24/7 EEG™ SubQ EEG data), across the 6 months of the study (‘correlation B’). Average difference between ‘correlation A’ and ‘correlation B’, across participants. Average difference between the number of AI‐driven automated seizure detections produced by EpiSight software and seizure counts estimated from rapid clinician review of annotated 24/7 EEG™ SubQ data, across participants. Clinician rating on the question ‘Overall, after review of annotated 24/7 EEG™ SubQ data for all participants, how would you rate your rapid clinician review?’, i.e., average score on a 10‐point Likert scale (‘the AI‐driven algorithm is very accurate compared to my rapid review’ (10) to ‘the AI‐driven algorithm is very inaccurate compared to my rapid review’ (1))

Secondary objectives	Corresponding endpoint(s)/outcome variable(s)
2 To investigate the clinical relevance of 24/7 EEG™ SubQ Solution	Change over time for average HCP questionnaires ‘Communication’, ‘Insight’ & ‘Potential of 24/7 EEG™’ scores at baseline, 2 months and 6 months
3 To investigate if the EEG data recorded with 24/7 EEG™ SubQ might be used to inform change(s) in clinical management plan for the participant	Proportion of investigators (i.e., treating clinicians) with a positive opinion, i.e., answer ‘Yes’ to the question ‘Did you adjust or change the clinical management plan during the past month?’ ‘(Yes/No)’. B Investigators rating of which data provided by 24/7 EEG™ SubQ Solution and/or clinical data led to change(s) of the clinical management plan: (i) Electrographic seizure, (ii) seizure type, (iii) seizure frequency, (iv) seizure severity, (v) cluster patterns, (vi) seizure periodicity, (vii) trigger factors, (viii) side effects of current anti‐seizure medication, (ix) lack of effects of current anti‐seizure medication, (x) non‐adherence to anti‐seizure medication, (xi) indication for adjusting or changing anti‐seizure medication, (xii) other. C Investigators rating of which adjustment(s) or change(s) to the epilepsy management plan initiated based solely or partly on data provided by 24/7 EEG™ SubQ Solution during the past month: (i) advice/education, (ii) referral, (iii) adjusting or changing anti‐seizure medication, (iv) prescription of other than anti‐seizure medication, (v) increased clinical follow‐up, (vi) changes in primary care, (vi) further examinations/test, (vii) other. D Investigators reason(s) for not changing the clinical management plan, i.e., answering ‘no’ on 3.A: (i) The patient is optimally treated (ii) Patient resistance to change in management plan despite medical, (iv) Insufficient data or information to adjust or change the clinical management plan) (v) other (free text). E Change over time for average HCP questionnaire ‘Management’ scores at baseline, 2 months and 6 months
4 To investigate participants' perception of the value of receiving feedback about seizure occurrences estimated from EEG data recorded with 24/7 EEG™ SubQ	Participants overall rating of the value of receiving feedback about seizures occurrences every month, across the full study period, from the EEG data recorded with 24/7 EEG™ SubQ i.e., average score on a 10‐point Likert scale (‘I found the feedback about when seizures had occurred according to the 24/7 EEG™ SubQ extremely helpful’) (10) to negative (‘I found the feedback about when seizures had occurred according to the 24/7 EEG™ SubQ extremely unhelpful’) (0))
5 To investigate the participants' acceptability of real‐world implementation with 24/7 EEG™ SubQ Solution	Raw score for IL01 Post market surveillance: Subject satisfaction form at 2‐months and change over time at 6‐months follow‐up
6 To investigate the participant adherence with 24/7 EEG™ SubQ Solution	Proportion of participants' percentage wear time with 24/7 EEG™ SubQ, across the study period of 6 months
7 To report the performance of 24/7 EEG™ SubQ Solution	Number of Device deficiencies during the study period (6 months)
8 To assess the quality of life with 24/7 EEG™ SubQ Solution	Change over time for average EQ‐5D‐5L scores at baseline, 2 months and 6 months. B Change over time for average Participant Weighted Quality of Life In Epilepsy (QOLIE‐31‐P) scores at baseline, 2‐months and 6 months. C Change over time for average Impact of epilepsy scores at baseline, 2‐months and 6 months. D Change over time for average Perceived Self‐Mastery Over Epilepsy scores at baseline, 2‐months and 6 months
9 To assess the health economic impact of epilepsy for participants that have used the 24/7 EEG™ SubQ Solution	Average Client Services Receipt Inventory (CSRI) scores at 6 months
10 To investigate the cost‐effectiveness of current practice and 24/7 EEG™ SubQ Solution	Quality‐adjusted life‐years (QALYs) score
11 To compare above participant reported outcome measurements (ePROM's) with historical controls obtained in previous study (10.1.4)	Endpoints 8 (A‐D) and 9 (A) compared with the 138 participants in SMILE study in the treatment‐a‐usual arm (xii)31), at baseline and 6 months follow‐up

**FIGURE 3:**
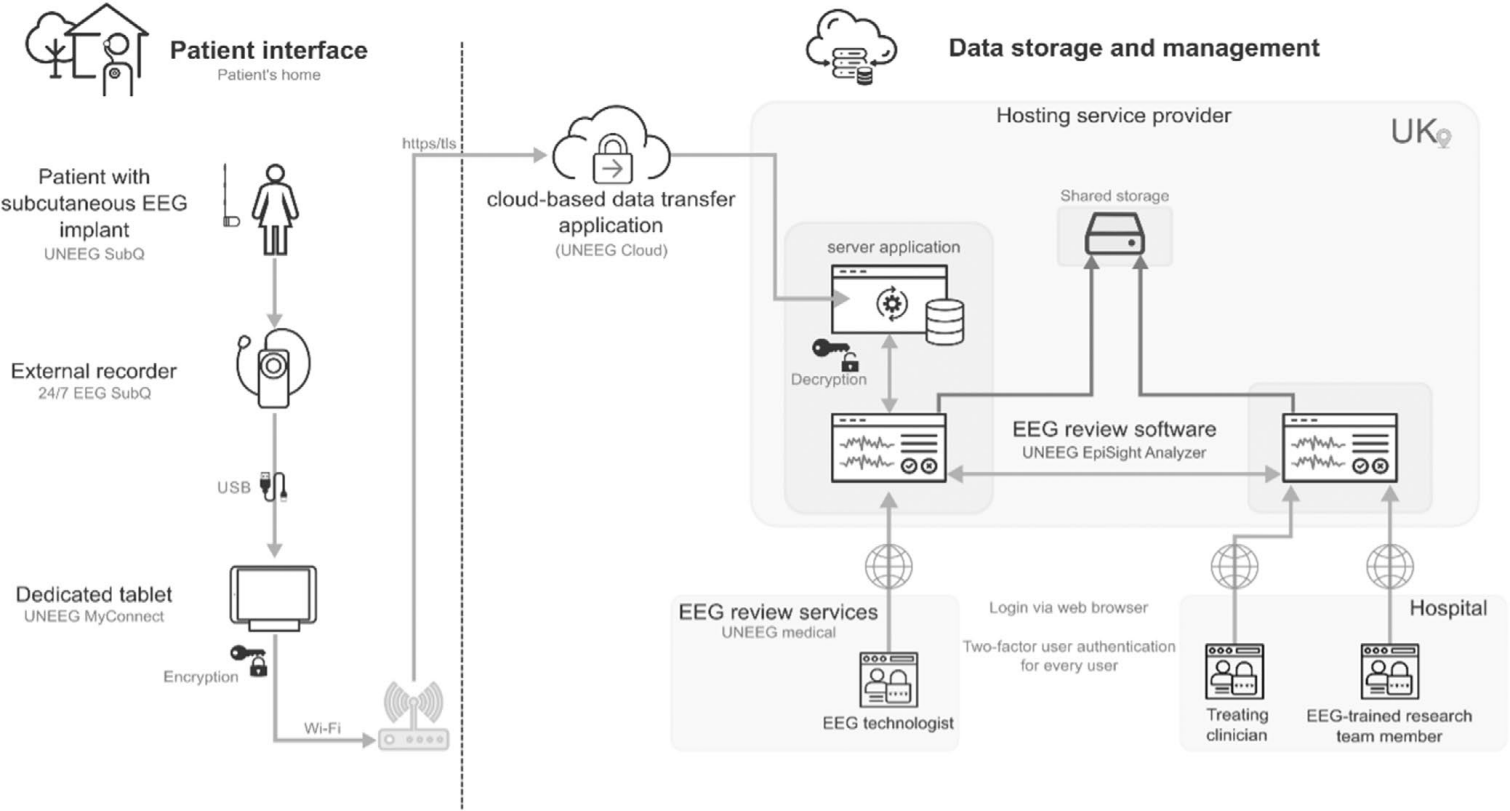
Data transfer flow of the 24/7 EEG SubQ solution using a 3rd‐party hosting service provider. Both the UNEEG EEG review services and the healthcare professionals have access to the data via a web browser.

Continuous 2 channel EEG data will be recorded from visit 3 to visit 5. To accommodate different local IT requirements, two data transfer configurations are possible whereby encrypted EEG data can either be stored within local hospital servers or stored within a third‐party cloud hosting service. The data pipeline for EEG is summarized in Figure 3. The participant will connect the external device daily via a USB cable to a tablet with a preinstalled application for data upload and recharging. Recorded EEG data will be automatically downloaded to the tablet, encrypted, and transferred via a cloud‐based data transfer application running in Microsoft Azure. The encrypted data in transit are pulled by an application (installed either at the servers of the local hospital IT network or in a 3rd‐party hosting service). Data are decrypted, stored, and made available to the investigators and treating clinicians in the UNEEG EpiSight Analyzer (EEG reading software).

Specialized third‐party contract research organization Clinigma (Copenhagen, Denmark) will conduct qualitative semi‐structured interviews with participants at baseline (between Visit 1 and Visit 2) and at exit (in the period of 14 days after their Visit 5). Timepoints are chosen to obtain participants' insights before implantation (baseline interview) and before explantation (exit interview). Clinigma will also conduct interviews with a nominated treating clinician from each of the sites.

All treating clinicians and investigators will undergo Good Clinical Practice (GCP) training.

### 3.2 Seizure detection

Continuous EEG monitoring will generate substantial data; therefore, an automated solution is required. Automated algorithms for seizure detection in multichannel EEG (reviewed by Baumgartner et al.^29^) show seizure detection sensitivity 59%–96% (median 81.4%) and false detection rate 0.08–5.38 per hour (median 0.3 per hour). The EEG analysis software AI algorithm that will be utilized (implemented within UNEEG EpiSight Analyzer) was trained using EEGs from 490 people (Temple University) and optimized on 24/7 EEG SubQ data from 32 participants. A deep neural network based on ResNet and a feature pyramid structure was designed to recognize seizures. Using 24/7 EEG SubQ data from nine PWE,^30^ a mean sensitivity of 89% and mean false positive rate 5.5/24 h was achieved. Hence, our algorithm is comparable to other multichannel EEG detection algorithms.^29^

Data will be screened by experienced neurophysiology technicians to remove suspected false positives prior to treating clinician review. Treating clinicians will be provided with annotated EEG data and a report summarizing the previous week's EEG data at weekly intervals and will have access to the continuous EEG recordings at any time.

## 4 OUTCOMES

The primary outcome measures of this study include: (A) Within participant correlation between seizure counts per month estimated from clinician review of annotated 24/7 EEG SubQ data (hybrid AI‐seizure detection algorithm and neurophysiology technician review) and ‘ground truth’ EEG seizure occurrences per month (derived from expert research team review of 24/7 EEG SubQ data); (B) Within participant correlation between seizure counts per month from participant reported seizure diary and ‘ground truth’; (C) Average difference between ‘correlation A’ and ‘correlation B’; (D) Average difference between the number of AI driven seizure detections and seizure counts estimated from rapid clinician review of annotated 24/7 EEG SubQ data (clinician inspection of data), across participants; and (E) Clinician rating of accuracy (compared to their own review) of annotated 24/7 EEG SubQ data.

A complete list of primary and secondary objectives with corresponding outcomes can be found in Table 1.

### 4.1 Statistical analysis of primary outcome measures

The research team will review raw EEG data from 24/7 EEG SubQ to identify seizures as the ‘ground truth’. We will assess the improvement in accuracy of seizure documentation using AI‐driven algorithm with rapid expert review applied to 24/7 EEG SubQ data (‘24/7 EEG SubQ detected seizures’), compared to using self‐reported participant diary alone. We will estimate for each participant the Spearman correlation between monthly diary report and ground‐truth, and the Spearman correlation between monthly 24/7 EEG SubQ detected seizures and ground truth. We will test the hypothesis that, across the cohort, the correlation between 24/7 EEG SubQ detected seizures and ground truth is higher than between the diary and ground truth. We will also calculate for diary and 24/7 EEG SubQ detected seizures the sensitivity, specificity, false positive rate, positive predictive value, and negative predictive value, for seizure detection. We will report using Standards for the Reporting of Diagnostic accuracy studies (STARD) principles.^31^

Feasibility and acceptability will be assessed by the proportion of participants who continue in the study for >3 months of 24/7 EEG SubQ data collection.

### 4.2 Statistical analysis of secondary outcome measures

Clinical relevance, changes in the clinical management plan for the participant, degree of confidence level of investigators initiating a change in their participant's clinical management plan, based on the input from the EEG data recorded with 24/7 EEG SubQ Solution, will be assessed by questionnaires.

Participant's reaction to receiving feedback on EEG data recorded with 24/7 EEG SubQ will be captured using an average score on a 10‐point Likert scale (positive (10) to negative (1)).

Adherence, acceptability, and performance will be assessed by: (1) Proportion of total study time (wear time) during which 24/7 EEG SubQ data are collected; (2) Questionnaires; (3) Proportion of Device Deficiencies.

In order to assess quality of life we will assess change over time for average EQ‐5D‐5L scores at baseline, 2 months and 6 months, change over time for average Participant Weighted Quality of Life In Epilepsy (QOLIE‐31‐P) scores at baseline, 2‐months and 6 months, change over time for average Impact of epilepsy scores at baseline, 2‐months and 6 months, and change over time for average Perceived self‐mastery over epilepsy scores at baseline, 2‐months and 6 months.

Costs and health outcomes will be estimated over a lifetime time‐horizon in order to consider longer‐term health and economic implications. Results of analysis will be presented in terms of cost per quality‐adjusted life‐year (QALY) gained. Average Client Services Receipt Inventory (CSRI) scores will be assessed at 6 months.

In order to compare our cohort with similar controls without 24/7 EEG SubQ, we will utilize historical controls from a recently completed study,^32^ which included 198 participants in the Treatment As Usual arm with ≥10 seizures/year. These participants were recruited similarly and completed four outcome measures that we will use at baseline and 6 months and CSRI.

In addition to the above, adverse events will be recorded throughout the study.

### 4.3 Statistical analysis methods

The accuracy of the two groups, i.e., (1) AI‐driven algorithm with rapid expert review applied to 24/7 EEG SubQ data vs. ground truth and (2) self‐reported participant diary vs. ground truth, will be compared in two ways: a correlation‐based comparison and an agreement‐based comparison. For the correlation‐based comparison, we will first estimate Spearman correlation coefficients for both groups to evaluate their monotonic relationship with the ground truth. To compare these relations between the two groups, we will use a Wilcoxon signed‐rank test. For the agreement‐based comparison, we will use the Bland–Altman method to allow us to calculate the mean difference and the limits of agreement to determine if one group consistently over‐r underestimates the ground truth compared to the other group.^33^

Questionnaire‐based data (see Table 1) will be assessed using different methods. For categorical data collected at three different time points (start, 2 months, and 6 months), the Chi‐squared test will be used to assess whether there are significant changes in the distribution of responses over time. Likert scale's and the CSRI will be assessed with descriptive statistics (e.g., mean, median, and frequency distributions) to summarize participant responses. For comparing the quality‐of‐life questionnaires (e.g., EQ‐5D, QOLIE‐31‐P) between the study cohort and historical controls, the Mann–Whitney U test will be used. Differences will be considered significant at *p* < 0.05.

## 5 OVERSIGHT AND MONITORING

Risk‐based monitoring will be carried out by a clinical study manager at UNEEG medical during the period of the investigation. As most source data in this investigation will be electronically archived, centralized monitoring will be performed continuously by the monitor. However, essential documents such as participant identification list and informed consent will be monitored on‐site.

The first monitoring visit will be an on‐site visit planned after the minimum of three and maximum of five participants have completed their first visit to identify any data entry errors (discrepancy between source data and eCRF), systematic errors caused at the site or by the participant, and provide any necessary help and assistance to the site. Most importantly, the aim will be to identify any issues that have or might have impacted the integrity of data and safety of participants early in the study for corrective actions to be implemented.

Remote monitoring visits will be held with an appointed investigator at each site, to follow up on recruitment, site progress, safety reporting, and on potential queries in the REDCap system or other outstanding issues. Remote monitoring visits will be planned every 4–8 weeks, or per 10 participants enrolled at the site.

The frequency of on‐site monitoring visits will be planned dependently on the outcome of the first on‐site visit, and continuous central monitoring and remote monitoring visits: if major issues are identified, a higher frequency of on‐site monitoring visits might be initiated.

### 5.1 Harms

All adverse events or device deficiencies will be reported within REDCap. The coordinating investigator at each site must assess all adverse events that occur on their site. All serious adverse events and serious adverse device effects must be reported to the principal investigator (PI) within 3 calendar days of the site becoming aware of the event. It is the responsibility of the PI to ensure that serious adverse events or serious adverse device effects are reported to national regulatory authorities immediately, but no later than 7 calendar days following the date of awareness by the PI.

If a suspicion of an unacceptable risk to participants develops during the clinical investigation, the PI will suspend the study while the risk is assessed. The PI will terminate the study if an unacceptable risk is confirmed. If monitoring or auditing of the clinical investigation identifies serious or repeated deviations at one of the participating investigation sites, the PI will suspend or terminate the investigation site.

## 6 DISCUSSION

Epilepsy is a multifaceted, common neurological disorder with a high burden of morbidity, mortality, and cost.^32^, ^33^, ^34^, ^35^, ^36^, ^37^, ^38^, ^39^, ^40^, ^41^ It is clear that poor seizure control with unexpected seizure occurrence results in a reduced quality of life for people with epilepsy and their families.^42^, ^43^ The development of a robust, accurate approach to seizure detection and recording is therefore of the utmost importance. Given the convenience of mobile devices, wearable technology, and the general desire of people with epilepsy for the integration of these advances into their healthcare,^41^, ^44^, ^45^, ^46^, ^47^ the study described here seems a logical next step. By enhancing the quality of information available to PWE, we may promote improved self‐management resulting in better self‐efficacy, adherence to treatment, and avoidance of seizure triggers.^48^, ^49^ In addition to this, we may also expedite impactful treatment decisions, which ordinarily could take multiple visits to the treating clinician.^50^, ^51^

Our study sets out to address a major issue in epilepsy management by pursuing alternative means of seizure monitoring to enable more reliably informed decision making. A particular strength of this study is the integration of the device into clinical practice without overly stringent inclusion criteria. This allows treating clinicians the freedom to make decisions based on their own interpretation of the solution's capabilities.

There are also some study limitations. Our sample size is small and may not be representative of the whole epilepsy population. Recruitment may prove challenging given the need for implantation and increased demands on the clinician. Clinicians will need to familiarize themselves with reviewing data, which could prove to be time consuming. If the AI‐driven algorithm provided by UNEEG proves to be inadequate from a clinician's perspective, the sheer amount of data analysis may not be feasible for busy clinicians. It also raises issues in the UK, where neurologists are not routinely EEG trained, which necessitates reliance on neurophysiologists, which is potentially quite complicated and labor intensive.

We will determine whether 24/7 EEG SubQ is feasible and acceptable to PWE and clinicians alike. REAL‐ASE hopes to establish new avenues to improve quality of life for PWE.

## AUTHOR CONTRIBUTIONS

MPR, JA, and JDH designed the study, coordinated its delivery, and wrote and amended the protocol for ethical approval. MM drafted the protocol for publication. PFV, MPR, JDH, ED, JA, JSW, and HH contributed to reviewing the protocol for publication. JD developed analytical methods for data subtraction. All authors have reviewed the manuscript and approved it for publication.

## FUNDING INFORMATION

The study is funded by the National Institute for Health Research (NIHR) through the Invention for Innovation (i4i) Challenge Awards call 12. This particular call required existing CE‐marked technology to be subjected to ‘real world’ study.

## CONFLICT OF INTEREST STATEMENT

Jonas Duun‐Henriksen and Jessica Agren are employees of UNEEG. Mark P. Richardson has received payment for ad‐hoc consultancy for UNEEG. Pedro F. Viana has received consultancy and travel fees from UNEEG. We confirm that we have read the Journal's position on issues involved in ethical publication and affirm that this report is consistent with those guidelines.

## ETHICAL APPROVAL

REAL‐ASE was approved by the Bromley Research Ethics Committee in July 2023, reference number 23/LO/0419.

## Data Availability

The data that support the findings of this study are available from the corresponding author upon reasonable request.
